# Ultraslow Myosin Molecular Motors of Placental Contractile Stem Villi in Humans

**DOI:** 10.1371/journal.pone.0108814

**Published:** 2014-09-30

**Authors:** Yves Lecarpentier, Victor Claes, Edouard Lecarpentier, Catherine Guerin, Jean-Louis Hébert, Abdelilah Arsalane, Abdelouahab Moumen, Xénophon Krokidis, Francine Michel, Oumar Timbely

**Affiliations:** 1 Centre de Recherche Clinique, Centre Hospitalier Régional de Meaux, Meaux, France; 2 Service de Gynécologie-Obstétrique, Centre Hospitalier Régional de Meaux, Meaux, France; 3 Department of Pharmaceutical Sciences, University of Antwerp, Wilrijk, Belgium; 4 Institut de Cardiologie, Hôpital de la Pitié-Salpêtrière, Assistance Publique-Hôpitaux de Paris, Paris, France; 5 INSERM U767, Université Descartes Paris 5, Sorbonne Paris Cité, Paris, France; Cardiological Center Monzino, Italy

## Abstract

Human placental stem villi (PSV) present contractile properties. *In vitro* mechanics were investigated in 40 human PSV. Contraction of PSV was induced by both KCl exposure (n = 20) and electrical tetanic stimulation (n = 20). Isotonic contractions were registered at several load levels ranging from zero-load up to isometric load. The tension-velocity relationship was found to be hyperbolic. This made it possible to apply the A. Huxley formalism for determining the rate constants for myosin cross-bridge (CB) attachment and detachment, CB single force, catalytic constant, myosin content, and maximum myosin ATPase activity. These molecular characteristics of myosin CBs did not differ under either KCl exposure or tetanus. A comparative approach was established from studies previously published in the literature and driven by mean of a similar method. As compared to that described in mammalian striated muscles, we showed that in human PSV, myosin CB rate constants for attachment and detachment were about 10^3^ times lower whereas myosin ATPase activity was 10^5^ times lower. Up to now, CB kinetics of contractile cells arranged along the long axis of the placental sheath appeared to be the slowest ever observed in any mammalian contractile tissue.

## Introduction

Contractile properties of the human placenta have been suggested for a long time. This was supported by numerous histological, morphological and biochemical arguments such as the presence of smooth muscle-like cells [1]–[4], the particular structure of the extra vascular part of human placental stem villi (PSV) [5]–[7], and the determination of both myosin ATPase activity and myosin content [8]–[10]. Contraction directed along the PSV long axis has been induced by means of KCl [11]–[14]. Mechanical behavior of PSV is reminiscent of that of smooth muscle insofar as they can be activated by KCl. However to date, isotonic mechanical properties explored over the entire load continuum have never been investigated in human PSV. The relationship between isotonic tension (T) and velocity (V) has been proven to be essential when its behavior is hyperbolic [15], [16]. In this case, characteristics of Hill's T-V relationship - i.e., asymptotes and curvature values - can be introduced in A. Huxley's equations [17] to calculate unitary force and kinetics of cross-bridge (CB) molecular motors. Of all theoretical models applied to contractile tissues, Huxley's CB model remains the most commonly accepted for calculating myosin kinetics in both striated and smooth muscles [17]–[19]. Nevertheless human PSV myosin kinetics remain totally unknown today. Recently the non muscle myosin type IIA (NMIIA) has been found to be localized in the extra vascular stromal tissue of human PSV while smooth muscle myosin types largely predominate within the vascular part [20]. Kinetics of the non muscle myosin type IIA cycle have been shown to be slow compared with those of other muscle myosins [21]. In the present study, using the A. Huxley formalism [17], we showed that the T-V relationship was hyperbolic and that kinetics of myosins cycling in isolated human PSV were dramatically slower by far than those previously reported for any contractile tissue. Comparisons between muscle myosin molecular characteristics were based on previous studies in which CB kinetics were determined by means of a similar method [18], [19], [22], [23].

## Materials and Methods

### Ethics Statement

Placenta were obtained from 40 pregnant women (age ranging from 18 to 35 years) undergoing delivery at the maternity of Meaux Hospital. Patients gave informed written consent with approval of the Local Ethical Committee, 2011,206, Direction Générale pour la Recherche et l'Innovation (DGRI) and Comité Consultatif sur le Traitement de l'Information en matière de Recherche dans le domaine de la Santé (CCTIRS), 2012.181. These institutions approved the study.

### Experimental Procedures

All pregnancies were normal. Exclusion criteria were as follows: delivery <37 weeks of gestational age, congenital malformations, chromosomal abnormalities, newborn with birth weight below the 10^th^ percentile [24], preeclampsia, and gestational diabetes. Characteristics of the pregnancies were (means ±SD): newborn weight (g): 3192±577; placental weight (g): 569±92; delivery gestational age (wk): 38±2; parity: 2.1±1.4; gravidity: 3.0±2.7.

The dissection protocol was standardized by cutting up a small piece of placenta (20-8-8 mm) from the middle part of a cotyledon. A PSV was carefully dissected. The PSV length at basal tone (Lo) was 10.2±2.6 mm and the PSV diameter was 1.5±0.3 mm. Thus PSV belonged to the type 1 category of the classification proposed by Demir [25]. Basal tone was the load imposed to the PSV which induced neither shortening nor lengthening of PSV before both KCl exposure and tetanus (0.97±0.41 mN.mm^−2^ in KCL; 0.82±0.34 mN.mm^−2^ in tetanus; p  =  0.31). PSV were horizontally positioned in the direction of the lever arm movement of the force transducer and disposed in a bath chamber containing a Krebs-Henseleit solution (in mM): 118 NaCl, 4.7 KCl, 1.2 MgSO_4_.7 H_2_O, 1.1 KH_2_PO_4_, 24 NaHCO_3_, 2.5 CaCl_2_.6H_2_O and 4.5 glucose, maintained at 22°C, pH 7.4, and bubbled with 95% O_2_-5% CO_2_. This induced a high O_2_ partial pressure in the bath which favored the oxygen diffusion into the PSV core. PSV were allowed to equilibrate for 30 min at basal tone.

Mechanics were assessed according to the longitudinal axis of the PSV. PSV were stimulated either by electrical tetanus (n = 20) (train period: 5s; train duration: 2s; stimulus frequency: 100 Hz; stimulus duration: 5 ms; tetanus duration: until reaching a plateau) or by KCl exposure (0.05 M) (n = 20). For each type of inotropic change (either tetanus or KCl exposure), the PSV was stimulated only one time. At the end of the study, the cross-sectional area (CSA in mm^2^) was calculated from the ratio of fresh PSV weight and length at basal tone (Lo). Diameter was calculated from CSA. Force (in mN) was normalized per CSA leading to tension (in mN. mm^−2^). Velocity was expressed in Lo.s ^−1^ and shortening length in % Lo.

### Electromagnetic Lever System

The electromagnetic lever system has been previously and extensively described [14].Briefly, the homemade lever system had three main functions: imposing a known force, measuring the load carried by the PSV and measuring the PSV displacement. For measurements on small PSV, a horizontal setup was preferred. The PSV was immersed in its Krebs-Henseleit solution and held by two miniature clips. One clip was attached to a fixed clamp, which position could be adjusted horizontally to bring the displacement transducer in range. The other clip was attached to the horizontally movable lever tip of a custom designed electromagnetic lever system. It was constructed from a suitable d'Arsonval panel meter whereby the pointer was replaced by a thin L shaped stainless steel tube acting as a lever. The lever system and its electronic control unit were designed to measure the shortening and loading of the PSV, to impose a load and to set the PSV initial length (and therefore the preload) of the PSV at rest. The displacement transducer consisted of a miniature Light Emitting Diode (LED) and a photodiode, mounted at both sides of a small vane attached to the lever. The light falling on the photodiode was proportional to the lever displacement. The resulting photocurrent was converted to a voltage by an operational amplifier, and its output signal was calibrated to a full scale of 10 V, corresponding to 1000 µm displacement at the lever tip. Since the electromagnetic torque is proportional to the current through the coil, the force at the tip of the lever could be set accurately by a voltage controlled current source. A servo loop was used for measuring the load carried by the PSV. The resting position of the lever could be controlled on a Graphical User Interface (GUI) as a set point. The system kept the lever at this position. It did so by adding/subtracting a correcting signal resulting in an adjustment of the current through the coil to counteract the force at the lever tip. As this voltage represented the force at the lever tip, this signal was filtered by a low pass filter delivering the force signal. It was calibrated to a full scale of 10 V corresponding to a maximal force of 20 mN. This system behaved as an isometric force transducer whereby the loop gain determined its stiffness and the PSV could only develop force at a constant length (isometric contraction). But the servo loop was designed in such a way the PSV could shorten when it developed more force than the total load imposed by the lever. Changing the set point could be compared by adjusting the position of a mechanical stop, and the servo circuit mimicked this mechanical stop holding the relaxed PSV at its initial length. The electrical stimulus was provided through two small platinum electrodes, attached to stainless steel rods arranged along both sides of the PSV. It was delivered by a galvanic isolated output stage.

The acquisition system was custom made and consisted of several blocks. The micro-processor firmware handled the commands from the host PC via an USB interface and sent A/D (analog to digital) converter data (displacement and force) to the host. Other commands set the stimulation parameters: period of train, number of pulses in a train, period and duration of stimulation pulses. A GUI on the host PC displayed a slider which enabled the user to adjust the set point position of the lever. Buttons and menus made it possible to select the sample rate and hence the duration of a single record. All data were displayed on the PC screen in real time and could be saved on the hard disk for further processing later on.

### Parameters of Contraction

**Figure 1:**
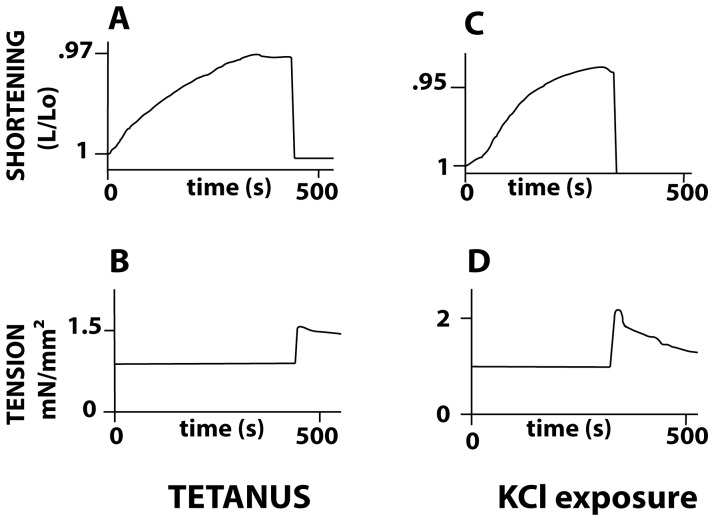
Changes in PSV contractile activity were induced by means of either electrical tetanus (panels A and B) or KCl exposure (panels C and D). Panels A and C: PSV shortening length versus time curves; Panels B and D: PSV tension versus time curves. Just after the onset of stimulus, a slow isotonic shortening at basal tone began, and reached a plateau. Basal tone was the load imposed to the PSV which induced neither shortening nor lengthening of PSV before both KCl exposure and electrical tetanus. PSV was then abruptly submitted to isometric conditions. After a brief overshoot due to the load clamp, PSV tension progressively decreased towards a plateau representing the total isometric tension.

Changes in contractile activity were induced by means of either electrical tetanus (Fig. 1, panels A and B) or KCl exposure (Fig. 1, panels C and D). Just after the onset of stimulus, a very slow isotonic shortening at basal tone began, and reached a plateau. PSV was then abruptly submitted to isometric conditions. After a brief overshoot due to the load clamp, tension decreased asymptotically towards a plateau corresponding to the total isometric tension. Total isometric tension was 167±98% of the basal tone after KCl exposure and 85±50% of the basal tone in tetanus (p = 0.03).

### Hyperbolic Behavior of the T-V Relationship

**Figure 2:**
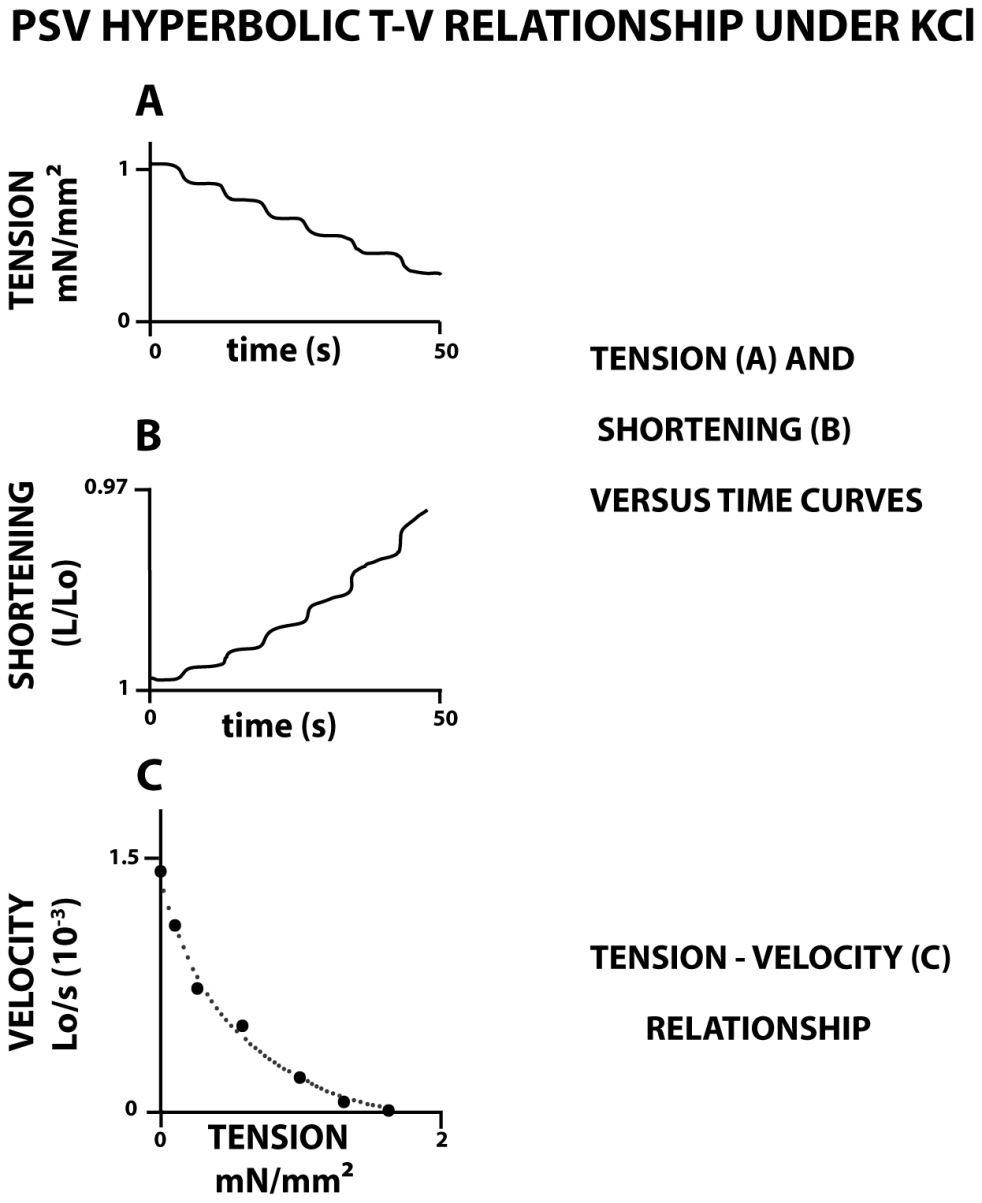
Hyperbolic Tension-Velocity (T-V) relationship under KCl exposure. In panel A, the isometric tension level T was progressively decremented by successive steps of 0.1 mN from maximum tension to zero-tension (Panel A: PSV tension versus time curve). In panel B, (PSV shortening length versus time curve), at each step, PSV shortened longitudinally at a given constant isotonic tension level (T) and constant velocity (V). The slope of the isotonic shortening length, i.e., the PSV shortening velocity, progressively increased when the isotonic tension (T) level decreased, until a maximum value Vo reached at zero-tension. In this example, the hyperbolic T-V relationship was derived from the velocity (V) of 7 isotonic afterloaded contractions, plotted against the isotonic tension level, according to the classic A.V. Hill equation (T+a) (V+b)  =  (To+a) b, where – a and – b are the asymptotes of the hyperbola [15]. The hyperbolic T-V relationship obtained under electrical tetanus was strictly similar (not represented).

To compare the respective inotropic effects of both tetanus stimulation and KCl exposure on the T-V relationship, a similar isometric tension level (To, Table 1) was chosen. To (mN. mm^−2^) was 1.39±.86 in electrical tetanus and 1.32±.97 after KCl exposure (p = 0.56). The isometric load level To was progressively decremented by successive steps of 0.1 mN until zero load (Fig. 2). At each step, PSV shortened longitudinally at isotonic tension level (T) and constant velocity (V). Vo is the maximum shortening velocity at zero-load. When T decreased, V increased. The T-V relationship was derived from the velocity (V) of 7 to 10 isotonic afterloaded contractions, plotted against the isotonic load level normalized per cross-sectional area. The T-V relationship was fitted with a hyperbola according to the classic A.V. Hill equation (T+a) (V+b)  =  (To+a) b, where – a and – b are the asymptotes of the hyperbola [15]. The G curvature of A.V. Hill's equation is equal to To/a = Vo/b, where To is the peak isometric tension, and Vo the maximum unloaded shortening velocity. Asymptote “a” was expressed in mN/mm^2^ and “b” in Lo/s. G is dimensionless.

**Table 1:** Mechanical parameters of human placental stem villi (PSV) and molecular myosin characteristics under electrical tetanus and KCl [0.05M].

	Electrical tetanus n = 20	KCl exposure n = 20	P
Vo; Lo.s^−1^	1.4±.0.4 (10^−3^)	1.3±.0.1 (10^−3^)	0.71
To; mN.mm^−2^	1.39±.86	1.32±.97	0.56
G	3.3±1.7	3.8±1.8	0.67
f_1_; s^−1^	.083±.066	.068±.050	0.66
g_1_; s^−1^	.044±.055	.027±.019	0.19
g_2_; s^−1^	.27±.14	.33±.24	0.16
π; pN	1.9±.3	2.0±.3	0.46
k_cat_; s^−1^	.005±.005	.003±.002	0.25
myosin content; nM.g^−1^	.145±.099	.130±.062	0.51
max.ATPase; nM. g^−1^.s^−1^	4.7±.1 (10^−4^)	4.8±.1 (10^−4^)	0.97

Vo: PSV maximum velocity at zero-load; Po: PSV peak isometric tension; G: curvature of the hyperbolic T-V relationship; CB attachment (f_1_) and detachment (g_1_ and g_2_) constants; π: CB unitary force; k_cat_: catalytic constant; max.ATPase: maximum myosin ATPase activity. Values are means ±SD. For all parameters presented in Table 1, there was no statistical difference between tetanus and KCl exposure by applying the unpaired t test and as attested by the p-values.

### A. Huxley Formalism; CB Unitary Force and CB Kinetics (see the Appendix)

A. Huxley's equations can be applied to both striated and smooth muscles as proposed in his princeps study [17]. This made it possible to calculate the rate constants for CB attachment (f_1_ in s ^−1^) and CB detachment (g_1_ and g_2_ in s ^−1^), maximum turnover rate of myosin ATPase under isometric conditions (kcat in s ^−1^), unitary force per single CB (˙πin pN), number (N) of active CBs per cross-sectional area (CSA), myosin content (in nM.g^−1^), and maximum myosin ATPase activity ( = k_cat_× myosin content) (in nM. g^−1^.s^−1^).

### Statistical Analysis

Data are expressed as means ±SD. Comparisons were made by using Student's unpaired t-test. One-way ANOVA was used to test for differences between KCl exposure and electrical tetanus stimulation; p-values <0.05 were required to rule out the null hypothesis.

## Results

The tension-velocity (T-V) relationship of isolated PSV was successively recorded after electrical tetanus and after KCl exposure. To allow comparisons between tetanus and KCl, a same level of isometric tension (To; mN/mm^2^) was imposed under the two conditions: To  = 1.39±.86 in tetanus and 1.32±.97 under KCl exposure (p = 0.56) (Table 1). The maximum unloaded shortening velocity (Vo) did not differ under these two conditions (p = 0.71) (Table 1). Tension, shortening length and shortening velocity were registered by decrementing several load levels from isometric condition to zero-load (Fig. 2 A, B and C). At any given level of isotonic load, PSV shortened at a constant velocity. We observed a hyperbolic relationship between isotonic load level and shortening velocity both in tetanus and KCl exposure (Fig. 2C). The characteristics of the hyperbola did not differ between tetanus and KCl: *a* = 0.49±0.34 mN/mm^2^ (tetanus) and 0.45±0.36 mN/mm^2^ (KCl exposure), (NS); *b* = 0.0007±0.0004 Lo.s^−1^ (tetanus) and 0.0005±0.0003 Lo.s^−1^ (KCl) (NS). This hyperbolic behavior between tension and velocity allows to apply Huxley's formalism [17]. This made it possible to determine myosin CB molecular characteristics. The following main parameters were determined under both tetanus and KCl and were presented in Table 1 (mean values ±SD and p-values): rate constants for CB attachment (f_1_) and CB detachment (g_1_ and g_2_), CB unitary force (˙π, myosin catalytic constant (k_cat_), myosin content and maximum myosin ATPase activity. None of these molecular parameters differed between tetanus and KCl conditions (see the p-values in Table 1).

## Discussion

For a long time, numerous histological and biochemical studies [1]–[4] indicated that the human placenta presented similarities with smooth muscle. This was corroborated by the description of the extra vascular part of human PSV [5]–[7], and the determination of the myosin content and myosin ATPase activity [8]–[10]. Importantly, Krantz and Parker, and Farley et al. have clearly demonstrated the contractile behavior of human PSV inducing contraction by mean of KCl exposure [11], [12]. Recently, tetanic electrical stimulation has been shown to induce human PSV shortening and increase in tension [14]. Moreover, PSV mechanics have never been assessed at various load levels, and molecular properties of human PSV myosin remain unknown. Activation of human PSV by either tetanus or KCl exposure led to similar characteristics of the T-V hyperbolic relationship and similar values of unitary myosin CB force, CB kinetics, myosin content and maximum ATPase activity (Table 1).

### Integrative Comparisons of CB Properties between Various Contractile Tissues

#### Comparisons of human PSV mechanics and CB kinetics with other mammalian contractile systems

Human PSV mechanics and myosin CB kinetics were compared with their counterparts in currently used smooth and striated (heart and skeletal) mammalian muscles [26]–[28]. In Table 2 are presented means ±SD of muscle mechanics and molecular CB myosin parameters in various smooth and striated muscles previously published from our laboratory and calculated with the same Huxley formalism [17], [22], [23]. Table 2 allows comparisons with data presented in Table1.

**Table 2:** Mechanical parameters of smooth and skeletal muscles and CB characteristics previously published in the literature [19], [22], [23].

	Uterus	Trachea	Heart	Soleus	EDL
Vo; Lo/s	.026±.015	.168±.047	3.25±.65	1.64±.50	5.81±1.00
To; mN/mm^2^	37±13	13.5±3.2	39±9.9	106±26	83±24
G	1.7±.3	2.9±.7	1.6±.2	6.6±1.1	3.7±.5
f_1_; s^−1^	2.5±1.9	8.9±2.4	242±57	38±11	242±55
g_1_; s^−1^	1.5±1.2	3.4±1.5	148±49	6.1±2.2	68±23
g_2_; s^−1^	5.7±4.1	33±9	560±124	331±98	1170±199
π; pN	1.6±.1	1.9±.1	1.7±.1	2.3±.1	2.1±.1
k_cat_; s^−1^	.17±.13	.42±.16	16.25±4.60	.92±.32	9.27±2.85
myosin content; nM/g	3.5±1.2	7.1±1.7	9.2±2.9	4.9±1.2	5.3±1.3
max.ATPase; nM/g/s	.58±.57	2.92±1.10	156±91	4.89±2.30	47±20

Vo: maximum velocity at zero-load; To: peak isometric tension; G: curvature of V-T relationship; CB attachment (f_1_) and detachment (g_1_ and g_2)_ constants; π: CB unitary force; k_cat_: catalytic constant; max. ATPase: maximum myosin ATPase activity. EDL, Soleus, Trachea, Uterus were stimulated under tetanic electrical stimulation and Heart under twitch electrical stimulation.

#### Comparisons with muscle parameters

Human PSV maximum shortening velocity (Vo) was the slowest ever recorded in any mammalian contractile structure, i.e., about 4500 times slower than that observed in fast striated muscles [29], [30] (Tables 1 and 2). In human PSV, Vo was about 100 times slower than that in tracheal smooth muscle and about 20 times slower than in human uterus smooth muscle [22], [23]. Isometric tension developed in human PSV was markedly lower than in other contractile tissues mainly due to the low myosin content (Tables 1 and 2).

#### Comparisons with the G curvature of the Hill hyperbola

The G curvature of the hyperbolic T-V relationship was of same order of magnitude whatever the contractile tissue (Tables 1 and 2). This explains why the CB unitary force (π) was also of the same order of magnitude whatever the contractile tissue, due to the relationship betweenπ and G, which is inherent to A. Huxley's equations (see the Appendix).

#### Comparisons with molecular myosin CB parameters

Compared with other fast striated mammalian contractile tissues, f_1_, g_1_ and g_2_ were in PSV about 3500, 2500 and 3500 times lower, respectively (Tables 1 and 2). In non muscle myosin type IIA (NMIIA), actin binding to myosin has been previously shown to be dramatically slow [21]. In human PSV, the very low value of the rate constant for CB detachment g_2_ accounted for the very low value of maximum unloaded shortening velocity (Vo) as g_2_ is proportional to Vo [17] (see the Appendix; g_2_ = 2 Vo/h). This linear relationship between Vo and g_2_ is intrinsic to the A. Huxley formalism. This relationship is of great interest because it links a microscopic molecular parameter (g_2_) to a macroscopic organ parameter (Vo). This makes it possible to apply statistical mechanics to contractile tissues [22]. The CB attachment f_1_ and detachment g_1_ rate constants dramatically change according to the muscle type but these changes occurred in a coordinated manner (Table 2. This was attested by the two ratios f_1_/g_1_ and g_2/_(f_1_+g_1_) which vary over a small range throughout muscle types and which are equal to the Hill hyperbola curvature G [17]. This explains the small changes in CB unitary force (π due to the fact that π is proportional to f_1/_(f_1_+g_1_) (see the Appendix). Thus among contractile tissues, the range of variations of myosin II kinetics was large, but their coordinated changes which are inherent to A. Huxley' equations, allowed to maintain a small variation range in CB single force (Appendix and Tables 1 and 2).

The site of the myosin II containing the ATPase activity is located on the head of the molecule itself [17], and thus the low myosin content in the human placenta partly accounted for the low myosin ATPase activity which is equal to the product of k_cat_ and myosin content. Maximum myosin ATPase activity was 10^5^ times lower in human PSV than in mammalian fast skeletal muscles [22], [29] (Tables 1 and 2). Myosin ATPase activity in human PSV was the slowest ever recorded in any mammalian contractile structure.

#### Comparisons of CB kinetics between human PSV and uterus smooth muscle

In Table 2, the muscle type presenting the slowest shortening velocity and myosin CB kinetics is the uterus smooth muscle. Thus, we compared CB kinetics in uterus (Table 2) and PSV (Table 1), both being stimulated under electrical tetanus. The following five parameters f_1_, g_1_, g_2_, k_cat_, and myosin ATPase activity were significantly lower in PSV than in uterus (p<0.0001 for each parameter). This means that myosin CBs cycled much more slowly in PSV than in uterus. Moreover, the unitary CB force (π) and the curvature (G) of the T-V hyperbolic relationship were higher in PSV (Table 1) than in uterus (Table 2) (p = 0.0021 and p = 0 0025, respectively).

#### Non muscle myosin

Although the vascular part of human PSV is composed of smooth muscle myosin, the extravascular part has been found to be mainly composed of non muscle myosin IIA [20]. Non muscle myosins II (NMII) like all myosin II molecules are hexamers composed of myosin heavy chain dimers and two pairs of myosin light chains (MLC). They can bind reversibly to actin filaments, hydrolyze ATP in an actin-activated process and thereby convert chemical energy into mechanical force and movement. Similar to other members of the myosin II family, NMII forms bi-polar filaments which are smaller than those observed in cardiac and skeletal myosin [31]. The regulation of both vertebrate non muscle and smooth muscle myosin II is through phosphorylation of the 20 kDa MLC. Non muscle myosins II are involved in various cellular functions, i.e., generation of cell polarity, cell division, cell migration, and cell-cell and cell-matrix adhesion [31], [32]. However, the contractile behavior of NMIIA in human PSV differs from those previously described [31]. In the present study, it was reminiscent of that of smooth muscles, as suggested by the observation of contractile processes induced by both KCl exposure and electrical tetanus, and by the presence of a hyperbolic tension-velocity relationship. Moreover in human PSV, relaxation has been shown to be induced by NO-donors [12], [14].

## Conclusions

The large tissue lattice of human PSV containing the contractile machinery was efficient enough to generate tension and shortening at extremely low velocity (Table 1). The ultraslow chemical kinetics and mechanical properties of PSV myosin molecular motors give the human placenta a rather special role among contractile systems, heralding the emergence of a new class of extremely ultraslow contractile tissue. Cross-bridge interactions in human PSV could contribute to local self-regulation, matching fetal intravillous blood flow to maternal intervillous blood status. Up to now, the myosin pattern present in the human placental PSV appears to be the slowest ever observed in any mammalian contractile tissue. The true role of PSV mechanical properties mimicking the behavior of both smooth and striated muscles remains to be determined in normal human PSV, as well as potential abnormalities in placental pathologies.

## Appendix

In his princeps study [17], A. Huxley has shown that his theoretical formalism could be applied to both striated and smooth contractile structures. According to A. Huxley's formalism [17], isotonic tension (P_Hux_) and rate of total energy release (E_Hux_) as a function of muscle velocity (V) are given by the following equation: 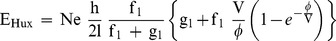


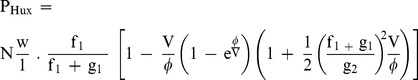
where f_1_ is the peak value of the rate constant for CB attachment; and g_1_ and g_2_ are the peak values of the rate constants for CB detachment; N is the cycling CB number per mm^2^ at peak isometric tension; w is the maximum mechanical work of a single CB (w/e = 0.75) [17]; and e is the free energy required to split one ATP molecule. According to A. Huxley's theory, one ATP is split per CB cycle, the standard free energy ΔG°'_ATP_ is nearby −60 kJ/mol, thus the value used for e was 10^−19^ J. The tilt of the myosin head relative to actin varies from 0 to h; f_1_ and g_1_ correspond to a tilt from 0 to h and g_2_ corresponds to a tilt>h;



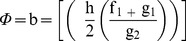



The molecular step size h is defined by the translocation distance of the actin filament per ATP hydrolysis, produced by the swing of the myosin head. In vitro single-head myosin produces approximately half the displacement (5 nm) of the in vivo double-head myosin (10 nm) during a unitary interaction with actin [33]. The parameter l is the distance between successive actin sites with which any myosin site can combine with actin. According to in vivo conditions and A. Huxley conditions (l>>h) [17], the values of h and l were h = 10 nm and l = 28.6 nm (close to the semi helicoidal turn of the actin filament). Calculations of f_1,_ g_1_, and g_2_ were given by the following equations [30].
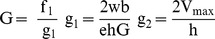


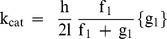


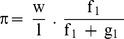



The number of active CBs/mm^2^ (N) is equal to the ratio of the peak isometric tension and the elementary CB force (π). The myosin content (nM.g^−1^) was calculated from the number of CB per g of tissue and the Avogadro number. The myosin ATPase activity was the product of k_cat_ and myosin content.
